# Effect of nerolidol on seizure and oxidative brain damage induced by pentylenetetrazole in mice

**DOI:** 10.1055/s-0046-1825524

**Published:** 2026-07-14

**Authors:** Seyed Hesam Hojjat, Fatemeh Rasouli, Farzaneh Shakeri

**Affiliations:** 1 School of Medicine, North Khorasan University of Medical Sciences, Bojnurd, Iran.; 2 Multiple Sclerosis Research Center, Neuroscience Institute, Tehran University of Medical Sciences, Tehran, Iran.; 3 Natural Products and Medicinal Plants Research Center, North Khorasan University of Medical Sciences, Bojnurd, Iran.; 4 Department of Physiology and Pharmacology, School of Medicine, North Khorasan University of Medical Sciences, Bojnurd, Iran.

**Keywords:** Sesquiterpenes, Seizures, Oxidative Stress, Pentylenetetrazole, Mice

## Abstract

**Background:**

Nerolidol, a natural sesquiterpene alcohol found in essential oils, has demonstrated antioxidant, anti-inflammatory, and neuroprotective properties. However, its effects on pentylenetetrazole (PTZ)-induced seizure and associated oxidative brain damage remain unclear.

**Objective:**

To evaluate the anticonvulsant effects of nerolidol on PTZ-induced seizures in mice and to investigate its impact on oxidative and nitrosative stress markers in the hippocampus and cortex.

**Methods:**

Male mice were divided into 5 groups: control, PTZ, and PTZ pretreated with nerolidol (25, 50, or 100 mg/kg, orally) 30 minutes before PTZ administration. Seizure activity was assessed by measuring latencies to minimal clonic seizures (MCSs) and generalized tonic-clonic seizures (GTCSs). After a behavioral evaluation, hippocampal and cortical tissues were analyzed for malondialdehyde (MDA), nitric oxide (NO) metabolites, superoxide dismutase (SOD), catalase (CT), and total thiol content.

**Results:**

Nerolidol significantly increased MCS and GTCS latencies compared with the PTZ group. In the hippocampus, all doses reduced MDA levels, while in the cortex this effect was observed at 50 and 100 mg/kg. Nitric oxide metabolites were decreased by the two higher doses in both brain regions. The PTZ-induced reductions in SOD and CT activities were reversed by nerolidol at all doses, and total thiol levels were restored at 50 and 100 mg/kg.

**Conclusion:**

Nerolidol exhibits anticonvulsant and neuroprotective effects on PTZ-induced seizures, likely mediated by reduced oxidative and nitrosative stress and enhancement of endogenous antioxidant defenses.

## INTRODUCTION

Epilepsy is a widespread and highly-debilitating neurological disorder affecting more than 70 million people worldwide. Its estimated prevalence is of 6.4 per 1 thousand individuals, with an annual incidence of ∼ 67.8 per 100 thousand people. The disorder exhibits a bimodal age distribution, with the highest risk observed in infants and older adults. Epilepsy is characterized by recurrent, largely-spontaneous seizures resulting from abnormal, excessive, or synchronous neuronal activity in the brain. 1

Despite the development of a considerable number of antiseizure drugs (ASDs), providing symptomatic seizure control by acting on ion channels within the neuronal membrane and neurotransmitter systems, approximately 1/3 patients fail to be controlled sufficiently. As ASD-related adverse events are common and ASD non-adherence was observed in almost 40% of cases in a cross-sectional study, 4 there has been a great interest in developing an antiepileptic medication capable of managing seizures effectively with the limited risk of behavioral comorbidities. 2 3 4 5

Growing evidence indicates a strong association between epilepsy and oxidative imbalance. Elevated levels of reactive oxygen species (ROS) have been reported in animal models of temporal lobe epilepsy induced by infectious insults. In addition, patients with epilepsy frequently exhibit increased circulating levels of malondialdehyde (MDA), a biomarker of enhanced lipid peroxidation. Due to the brain's high oxygen consumption and rich lipid composition, it is particularly susceptible to oxidative damage. Furthermore, impaired activity of endogenous antioxidant enzymes, such as superoxide dismutase (SOD), can exacerbate mitochondrial oxidative stress, thereby contributing to the initiation and persistence of epileptic seizures. 6 7 8

Previous studies 9 10 have demonstrated that the coadministration of antioxidants with approved ASDs can effectively attenuate oxidative damage and associated behavioral impairments in pentylenetetrazole (PTZ)-sensitized mice. Plant-based therapies are frequently used in epilepsy management due to their antioxidant properties and relatively low side-effect profile. This has stimulated international interest in identifying and developing novel herbal compounds with potential antiseizure activity. 11

Nerolidol is a prominent bioactive constituent commonly extracted from the essential oils of diverse plant species, including *Canarium schweinfurthii* , *Amaranthus retroflexus* , *Piper claussenianum* , and *Baccharis dracunculifolia* , 12 13 14 15 which presents a wide spectrum of pharmacological properties, including antioxidant, 16 anti-inflammatory, 17 antinociceptive, 18 anticancer, 19 antiulcer, 20 anxiolytic, 21 and anticonvulsant 22 activities. Studies 23 using zebrafish models of PTZ-induced seizures have demonstrated nerolidol's potential to mitigate convulsive behaviors. Subsequent research in murine models has further confirmed its ability to reduce seizure susceptibility and oxidative stress, highlighting nerolidol as a promising multifunctional agent for epilepsy research. 22

Given that epilepsy is a common neurological disorder with a significant global burden, and considering its association with oxidative stress and the limitations of the current treatments, there is growing interest in herbal medications such as nerolidol, which may yield reduced side effects and promising therapeutic efficacy. While previous studies have assessed the anticonvulsant and antioxidant properties of nerolidol in PTZ-induced kindling models, its role in specific brain regions such as the cortex and hippocampus at different doses by evaluating particular oxidative stress markers remains unresolved. Accordingly, the present investigation primarily aimed to determine the effect of nerolidol on seizure and oxidative brain damage induced by PTZ in mice to enhance our understanding of nerolidol as a unique agent for seizure control.

## METHODS

### Drugs and chemicals

Nerolidol, PTZ (Sigma-Aldrich Corporation), urethane (Alfasan Diergeneesmiddelen B.V.) were procured to conduct the present study. Additional chemical compounds, including dimethyl sulfoxide (DMSO), thiobarbituric acid (TBA), pyrogallol, and 2, 2'-dinitro 5, 5′-dithiodibenzoic acid (DTNB) were purchased from Merck KGaA. The Greiss reagent kit was obtained from Betagen.

### Animals

Twelve-week-old male Naval Medical Research Institute (NMRI) mice, each weighing between 20 and 30 g, were obtained from the North Khorasan University of Medical Sciences animal facility. They were housed under consistent laboratory conditions, featuring a regulated 12-hour light/dark cycle, ambient temperatures maintained at 21–22°C, and mean relative humidity of 55 ± 5%. Standard rodent chow and water were available without restriction. All the procedures were performed according to the National Institutes of Health's Guide for the Care and Use of Laboratory Animals, and they were approved by the North Khorasan University of Medical Sciences Ethics Committee (Protocol number: IR.NKUMS.AEC.1403.001).

### Experimental design

Nerolidol was dissolved in DMSO followed by dilution with saline to achieve the desired concentrations, and the final concentration of DMSO did not exceed 0.5%. The animals in the control and PTZ-only groups received an equivalent dose of the vehicle solution.

To induce seizures in mice, PTZ (100 mg/kg), a well-established gamma-aminobutyric acid type A (GABA-A) receptor antagonist, was administered intraperitoneally 30 minutes after nerolidol or the saline treatment. The animals were then placed individually in Plexiglas cages (measuring 30 × 30 × 30 cm), and their behaviors were monitored and recorded for 60 minutes. Seizure activity was evaluated based on the latency to minimal clonic seizures (MCSs) and generalized tonic-clonic seizures (GTCSs). 24 The MCSs were defined as continuous seizure activity progressing from mild myoclonic jerks to clonus of the face and forelimbs without loss of the righting reflex, while GTCSs were characterized by clonic movements of the limbs with loss of the righting reflex followed by full tonic extension of both forelimbs and hindlimbs.

A total of 50 mice were assigned to 5 different experimental groups as follows, with each group consisting of 10 animals based on previous studies 25 employing PTZ-induced seizure models and oxidative stress assessments: Group I – saline-injected control group; group II – PTZ-injected group; group III – 25 mg/kg nerolidol-injected group; group IV – 50 mg/kg nerolidol-injected group; and group V – 100 mg/kg nerolidol-injected group. The doses of nerolidol (25, 50, and 100 mg/kg) were selected based on previous experimental works. 16 26

### Tissue collection and sample preparation for oxidative stress assessment

Following the completion of the behavioral assessments, the animals were deeply anesthetized with urethane and subsequently humanely euthanized in accordance with approved institutional ethical guidelines. The brains were rapidly removed, rinsed in ice-cold phosphate-buffered saline (PBS) to eliminate blood contamination, and placed on an ice-cold dissection surface to preserve tissue integrity prior to the biochemical analyses.

The hippocampus and cerebral cortex were anatomically identified and dissected based on established mouse brain landmarks, following the Paxinos and Franklin's *The Mouse Brain in Stereotaxic Coordinates* , 5th edition (2019). 27 The hippocampus was isolated from the medial temporal region (approximately 1.3–2.5 mm posterior to the bregma, 1.0–2.0 mm lateral, and 1.2–2.0 mm ventral), while the cerebral cortex was dissected from the dorsal surface of the brain after the removal of the subcortical structures (∼ 0.0–2.0 mm posterior to the bregma, 0.5–2.0 mm lateral, and 0.0–1.0 mm ventral).

Tissues were collected bilaterally from both hemispheres and pooled for each animal to minimize inter-hemispheric variability. The isolated cortical and hippocampal tissues were homogenized separately on ice in chilled 10% (w/v) PBS (pH: 7.4). Homogenates were centrifuged at 10 thousand × g for 15 minutes at 4 °C, and supernatants were collected for the biochemical analyses. The resulting homogenates were used for the biochemical assessment of oxidative stress markers, including MDA, nitric oxide (NO), SOD, catalase (CT), and total thiol content. 28

### Lipid peroxidation assay

The MDA levels, used as an indicator of lipid peroxidation, were determined by MDA's reaction with TBA to form a red-colored complex. The homogenized tissue samples were mixed with a TBA/trichloroacetic acid (TCA)/hydrochloric acid (HCl) reagent and heated in a water bath for 40 minutes. After cooling, the samples were centrifuged at 1 thousand × g for 10 minutes, and the absorbance of the supernatant was measured at 535 nm. The MDA concentration was calculated using the following equation:



### NO level assay

The NO levels were assessed using the Griess reagent. Homogenates were mixed with the reagent and incubated at room temperature for 10 minutes. Absorbance was recorded at 540 nm, and nitrite concentration was quantified in µmol/g based on a sodium nitrite standard curve.

### Antioxidant enzymes activity assay

The SOD activity, expressed in units per gram of tissue (U/g tissue), was determined by SOD's ability to inhibit pyrogallol oxidation, measured at 560 nm. The assay was performed at a mean controlled temperature of 25 ± 1°C. The CT activity, expressed in U/g tissue, was measured by monitoring the decomposition of hydrogen peroxide at 240 nm.

### Total sulfhydryl group/total thiol (SH) content assay

Total thiol content was evaluated using DTNB, which reacts with thiol groups to form a yellow complex. Briefly, 1 mL of Tris- ethylenediaminetetraacetic acid (EDTA) buffer (pH: 8.6) was added to 50 µL of the homogenate, and the absorbance was read at 412 nm (A _1_ ). Then, 20 µL of DTNB reagent was added, and after 15 minutes at room temperature, the absorbance was measured again (A _2_ ). A blank absorbance (B) of the DTNB solution was also recorded. Total thiol concentration (mM) was calculated using the following formula:



### Statistical analysis

Data are presented as mean ± standard error of the mean (SEM). Prior to the inferential analysis, data normality was assessed using the Shapiro–Wilk test, and the homogeneity of the variances was evaluated using the Levene's test. As all datasets met the assumptions required for the parametric analysis ( *p* > 0.05), and the statistical differences among groups were analyzed using one-way analysis of variance (ANOVA) followed by the Tukey's post-hoc test for multiple comparisons. Values of *p* < 0.05 were considered statistically significant.

## RESULTS

### Effect of nerolidol on the seizure severity and latency in PTZ-treated mice

All groups except the control, which did not receive PTZ, exhibited MCSs and GTCSs following the administration of 100 mg/kg of PTZ.

**Figure 1:**
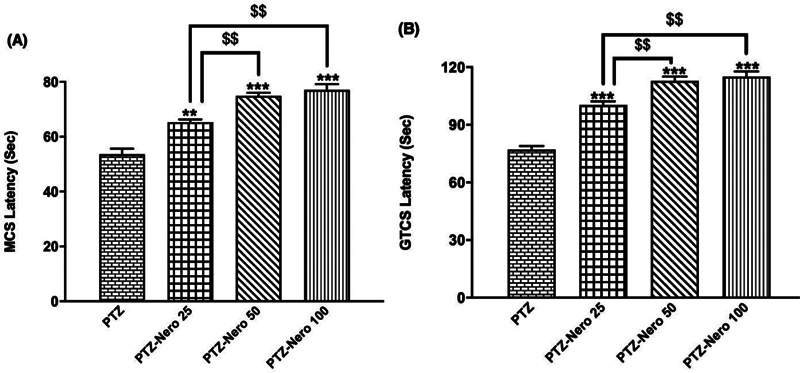
Notes: * *p* < 0.05; ** *p* < 0.01; and *** *p* < 0.001, as compared with the PTZ group; and ^$$^ *p* < 0.01 as compared with the PTZ-Nero 25 group. The effects of 25, 50 and 100 mg/kg of nerolidol on latencies to minimal clonic seizures (MCSs) ( **A** ) and generalized tonic–clonic seizures (GTCSs) ( **B** ) start after a single injection of 100 mg/kg of pentylenetetrazol (PTZ).

The one-way ANOVA analysis revealed a significant difference in MCS latency among the groups. Specifically, the groups pretreated with nerolidol showed significantly longer MCS latencies compared with the PTZ-only group. The nerolidol doses of 25, 50, and 100 mg/kg produced significant, dose-related increase in MCS latency ( *p* < 0.01 to *p* < 0.001) ( Figure 1A ).

Similarly, the one-way ANOVA indicated a significant difference in GTCS latency among the groups. Nerolidol at all 3 doses significantly increased the GTCS latencies ( *p* < 0.001) compared with the PTZ group, and the highest dose resulted in a significantly greater latency ( *p* < 0.01) ( Figure 1B ).

### Impact of nerolidol on brain lipid peroxidation levels

**Figure 2:**
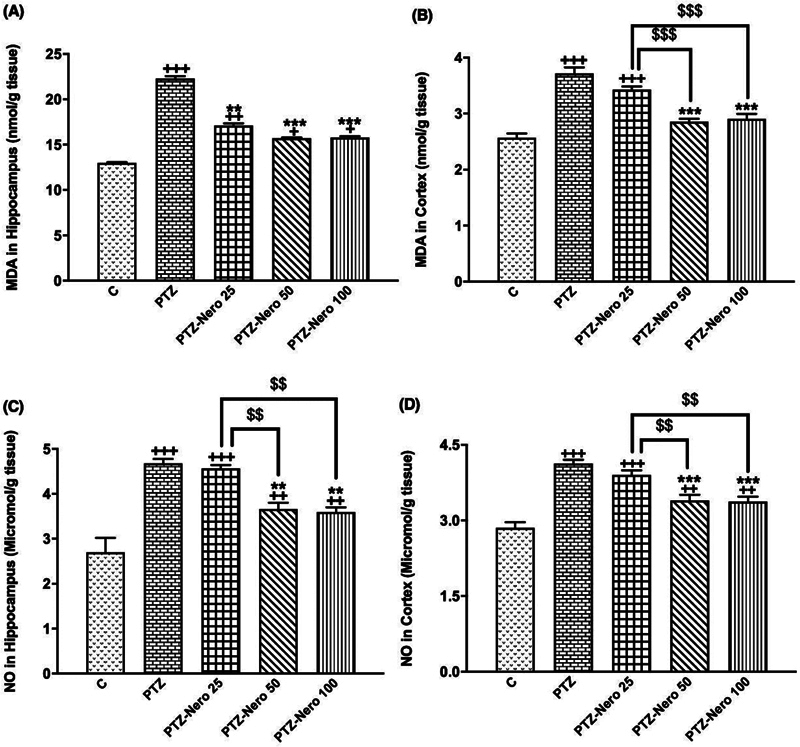
Notes: ^+^ *p* < 0.05; ^++^ *p* < 0.01; and ^+++^ *p* < 0.001, as compared with the control group; ** *p* < 0.01 and *** *p* < 0.001, as compared with the PTZ group; and ^$$^ *p* < 0.01 and ^$$$^ *p* < 0.001, as compared with the PTZ-Nero 25 group. The effects of 25, 50 and 100 mg/kg of nerolidol on malondialdehyde (MDA) and nitric oxide (NO) metabolites in hippocampal ( **A,C** ) and cortical ( **B,D** ) tissues after a single injection of 100 mg/kg of PTZ.

The levels of MDA, an indicator of lipid peroxidation, were significantly elevated in the hippocampal and cortical regions of the PTZ-treated group compared with the control group ( *p* < 0.001 for both; Figures 2A,B ). The administration of nerolidol prior to PTZ exposure significantly reduced hippocampal MDA concentrations across all tested doses compared with the PTZ group ( Figure 2A ; *p* < 0.01 to *p* < 0.001). However, no statistically significant differences in hippocampal MDA levels were detected among the PTZ-nero 25, PTZ-nero 50, and PTZ-nero 100 groups ( Figure 2A ).

In cortical tissues, MDA levels in the PTZ-nero 50 and PTZ-nero 100 groups did not differ significantly from control values, indicating near-normalization of lipid peroxidation at these doses. Pretreatment with nerolidol at 50 and 100 mg/kg significantly lowered MDA levels compared with the PTZ group, with greater effects observed at higher doses ( *p* < 0.001), while the 25 mg/kg dose showed no significant effect ( Figure 2B ).

As illustrated in Figures 2A,B , MDA concentrations in the hippocampus across all treatment groups, as well as in the cortex of the PTZ-nero 25 group, remained significantly higher than those in the control group ( *p* < 0.05 to *p* < 0.001).

### Impact of nerolidol on NO levels in brain tissue

The PTZ-treated group exhibited significantly elevated levels of NO metabolites in the hippocampal and cortical brain regions compared with the control group ( *p* < 0.001 for both; Figures 2C,D ).

Pretreatment with nerolidol at the medium (50 mg/kg) and high (100 mg/kg) doses led to a significant reduction in NO concentrations in both regions relative to the PTZ group ( *p* < 0.01 to *p* < 0.001). In contrast, the lowest dose (25 mg/kg) showed no significant effect on NO levels ( Figures 2C,D ).

As depicted in Figures 2C,D , NO metabolite levels in the hippocampus and cortex were significantly lower in the 50 and 100 mg/kg nerolidol groups when compared with the PTZ-nero 25 group ( *p* < 0.01 for all comparisons).

Despite this reduction, pretreatment with nerolidol at any of the tested doses did not fully normalize NO levels. The concentrations of NO metabolites in all nerolidol-treated groups remained significantly elevated in both brain regions when compared with the control group ( *p* < 0.01 to *p* < 0.001).

### Changes in SOD activity

**Figure 3:**
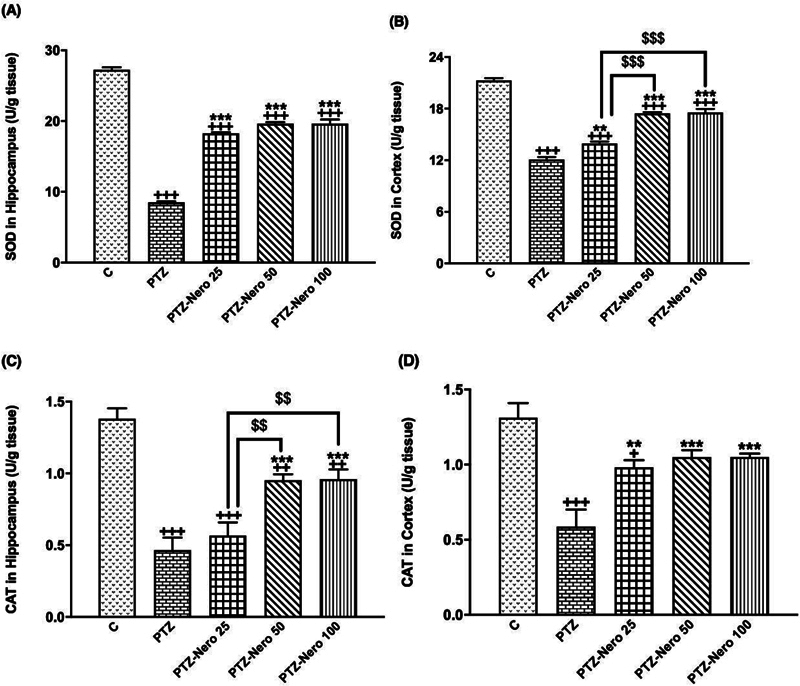
Notes: ^+^ *p* < 0.05. ^++^ *p* < 0.01; and ^+++^ *p* < 0.001, as compared with the control group; *** *p* < 0.001, as compared with the PTZ group; and ^$$^ *p* < 0.01; and ^$$$^ *p* < 0.001, as compared with the PTZ-Nero 25 group. The effects of 25, 50 and 100 mg/kg of nerolidol on superoxide dismutase (SOD) and catalase (CT) in hippocampal ( **A,C** ) and cortical ( **B,D** ) tissues after a single injection of 100 mg/kg of PTZ.

In the PTZ-treated group, SOD enzymatic activity was significantly reduced in the hippocampus and cortex compared with the control group ( *p* < 0.001; Figures. 3A,B ). Pretreatment with nerolidol at all tested doses led to a significant enhancement of SOD activity in the hippocampus relative to the PTZ group ( *p* < 0.001; Figure 3A ).

Similarly, cortical SOD activity was significantly increased across all nerolidol doses when compared with the PTZ group ( *p* < 0.01 to *p* < 0.001; Figure 3B ). Notably, SOD activity in the cortex was higher in the PTZ-nero 50 and PTZ-nero 100 groups than in the PTZ-nero 25 group ( *p* < 0.001; Figure 3B ). Despite these improvements, SOD activity in the hippocampus and cortex of all nerolidol-treated groups remained significantly lower than those observed in the control group ( *p* < 0.001).

### Changes in CT activity

The CT activity in the hippocampal and cortical regions exhibited a marked reduction ( *p* < 0.001; Figures 3C,D ) in the PTZ-treated group compared with the controls. The administration of 50 and 100 mg/kg of nerolidol before PTZ injection elevated CT activity within the hippocampal region compared with the PTZ-only group ( *p* < 0.001), but the dose of 25 mg/kg was not effective ( Figure 3C ). In addition, the CT activity in the hippocampal and cortical regions in groups treated with the medium and high doses of nerolidol was higher than that of the PTZ-nero 25 group ( *p* < 0.01).

In cortical tissue, CT activity in the PTZ-nero 50 and PTZ-nero 100 groups did not differ significantly from control values, indicating partial normalization of enzymatic antioxidant capacity at these doses. Pretreatment with nerolidol at all doses in the cortical region resulted in a significant increase in CT activity in comparison with the PTZ group ( *p* < 0.01 to *p* < 0.001; Figure 3D ). Compared to the control group, CT activity in the hippocampus were notably diminished in the PTZ-nero 25, PTZ-nero 50, and PTZ-nero 100 groups, as well as in the cortical region in the PTZ-nero 25 group ( *p* < 0.05 to *p* < 0.001, Figure 3C,D ).

### Impact of nerolidol on brain total thiol level

**Figure 4:**
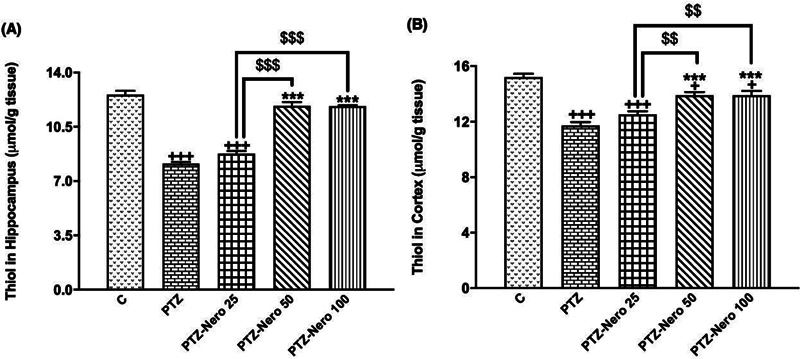
Notes: ^+^ *p* < 0.05; ^+++^ *p* < 0.001, as compared with the control group; *** *p* < 0.001 ,as compared with the PTZ group; and ^$$^ *p* < 0.01; and ^$$$^ *p* < 0.001, as compared with the PTZ-Nero 25 group. The effects of 25, 50 and 100 mg/kg of nerolidol on total thiol content in hippocampal ( **A** ) and cortical ( **B** ) tissues after a single injection of 100 mg/kg of PTZ.

Assessment of total thiol concentrations revealed a substantial decline in the hippocampal and cortical regions of the PTZ-treated group compared with the controls ( *p* < 0.001; Figure 4 ). The administration of nerolidol at doses of 50 mg/kg and 100 mg/kg prior to PTZ exposure resulted in significantly-elevated thiol levels in these brain areas relative to the PTZ group ( *p* < 0.001). In contrast, treatment with 25 mg/kg of nerolidol did not yield a statistically significant change.

In the hippocampus, the total thiol concentrations in the PTZ-nero 50 and PTZ-nero 100 groups did not differ significantly from control values, highlighting that these doses restored thiol levels to near-control levels.

Further comparisons indicated that thiol content in the hippocampus and cortex was notably higher in the PTZ-nero 50 and PTZ-nero 100 groups than in the PTZ-nero 25 group ( *p* < 0.01 to *p* < 0.001). However, thiol concentrations in the cortex of the PTZ-nero 25 group, as well as in all the hippocampus of the other groups treated with nerolidol, remained significantly below the levels observed in the control group ( *p* < 0.05 to *p* < 0.001).

## DISCUSSION

Epilepsy is a common neurological disorder resulting from an imbalance between excitatory and inhibitory neurotransmission. Its pharmacological management remains challenging because ∼ 30% of the patients develop pharmacoresistance, and the clinical use of current antiepileptic drugs is limited by unavoidable adverse effects. 29

Increasing evidence highlights the pivotal contribution of oxidative stress to the onset and progression of epileptic seizures. Alterations in ROS, reactive nitrogen species (RNS), and NO signaling pathways promote neuronal injury and impair cognitive, learning, and memory functions, underscoring the need for anticonvulsant strategies that also possess antioxidant capacity. Therefore, compounds derived from essential oil, such as nerolidol, represent promising alternative therapeutic candidates. 16 In the present study, the PTZ-induced seizure model was used to evaluate the anticonvulsant and antioxidant potential of nerolidol.

A well-established GABA-A receptor antagonist, PTZ is widely used to evaluate novel anticonvulsant agents in experimental models, 30 and it was administered at a dose of 100 mg/kg to induce epileptic seizures in the current study. The administration of PTZ produced rapid onset of MCSs and GTCSs, whereas pretreatment with nerolidol significantly delayed the onset of both seizure types compared with the PTZ-only group, indicating a protective anticonvulsant effect.

This behavioral protection was accompanied by biochemical evidence of oxidative imbalance. In contrast to the control animals, PTZ-treated mice exhibited a marked increase in lipid peroxidation and nitrosative stress, as evidenced by elevated MDA and NO levels, together with a significant reduction in antioxidant enzyme activities (SOD and CT), confirming the induction of oxidative stress. These findings support the concept that excessive free-radical production overwhelms the endogenous antioxidant defenses and promotes neuronal oxidative injury. 31 32

Nitric-oxide signaling plays a complex and context-dependent role in seizure regulation. Alterations in the NO pathway are considered a cornerstone of convulsive seizure development through the modulation of glutamatergic and GABAergic neurotransmission in PTZ-induced models. Under physiological conditions, basal NO levels may suppress seizure activity, whereas excessive NO during pathological states can facilitate neuronal hyperexcitability. 33

These controversial effects of NO, which is a key neurotransmitter involved in synaptic plasticity, neuronal excitability, and epileptic activity, obscure whether NO exerts pro- or anticonvulsant actions. 34 35

In research conducted by Taskiran and Tastemur 36 exploring the dual role of NO in seizure regulation, inhibition of NO synthase intensified limbic seizures induced by 60 mg/kg PTZ while protecting against tonic seizures at 80 mg/kg. These findings emphasize the dose-dependent and seizure-type-specific influence of NO signaling. 36

Accordingly, the observed decrease in NO contents following nerolidol treatment should be interpreted cautiously as an indicator of reduced seizure associated oxidative stress rather than a direct NO-mediated anticonvulsant mechanism.

Antioxidant therapies are increasingly recognized for their ability to mitigate seizure-induced oxidative injury. The elevated MDA levels and reduced antioxidant capacity observed in epilepsy patients may be partially corrected by the antiepileptic treatment. 37 38

Notably, approved ASDs such as valproate have been reported 39 to attenuate oxidative alterations in experimental seizure models; however, their primary mechanisms involve ion-channel modulation and neurotransmitter regulation, and their clinical use may be limited by toxicity and adverse effects.

Given the therapeutic potential of medicinal plants in epilepsy management, nerolidol, a sesquiterpene alcohol with antioxidant properties, may represent an alternative agent to enhance treatment efficacy. 40

In the current investigation, the administration of nerolidol significantly increased MCS and GTCS latencies compared with the PTZ group. These findings are consistent with prior research by Kaur et al., 26 who reported suppression of seizure progression and oxidative stress in PTZ models following nerolidol treatment. Additionally, behavioral studies in zebrafish models 23 have shown that nerolidol increases seizure latency while reducing seizure severity.

Supporting these findings, neurodegeneration research 41 indicates that nerolidol mitigates oxidative and neuroinflammatory injury. In the present study, pretreatment with nerolidol enhanced antioxidant defenses (SOD, CT, and total thiol content) while reducing MDA and NO levels, with more pronounced effects observed at higher doses. The limited efficacy of the lowest dose aligns with a dose-dependent protective profile.

The ability of the nerolidol to attenuate the oxidative stress during PTZ-induced seizure is consistent with the findings by Nogueira Neto et al. 16 who reported that nerolidol decreased MDA and NO levels and increased the activities of SOD and CT relative to the negative control group.

Although several antiepileptic drugs suppress seizure activity without adequately addressing oxidative injury, 42 43 nerolidol has demonstrated antiseizure efficacy and significant attenuation of oxidative and nitrosative stress. Although epileptogenesis was not directly assessed, the combined behavioral and biochemical findings suggest that nerolidol may provide neuroprotective benefits by limiting seizure-associated neuronal damage.

Despite these promising findings, further studies are required to elucidate the molecular mechanisms underlying nerolidol's effects, compare its efficacy with established anticonvulsants, and evaluate its long-term therapeutic potential following chronic administration.

In conclusion, the findings derived from the current investigation enhance our understanding of the anticonvulsant and antioxidant effects of nerolidol in PTZ-induced seizure, which require further preclinical studies evaluating its efficacy by focusing on the molecular mechanisms of how nerolidol possesses antioxidant and antiepileptic activities.
